# Wilfully submitting to and publishing in predatory journals - a covert form of research misconduct?

**DOI:** 10.11613/BM.2021.030201

**Published:** 2021-08-05

**Authors:** Nicole Shu Ling Yeo-Teh, Bor Luen Tang

**Affiliations:** 1 Research Compliance and Integrity Office, National University of Singapore, Singapore; 2 Department of Biochemistry, Yong Loo Lin School of Medicine, National University of Singapore, National University Health System, Singapore

**Keywords:** falsification, predatory publishing, predatory journals, scientific misconduct, research misconduct

## Abstract

A predatory journal could be provisionally defined as one masquerading as a genuine academic publication but offer little, if any, rigorous peer review. Predatory journals or publishers place a focus on maximising financial profit, as opposed to regulated dissemination of scientific advancements. As a result, authors can often get their work published in such journals with little scrutiny on quality. Although generally warned against and discouraged, universally practiced sanctions against researchers’ submission to and publication in predatory journals are not common. Predatory publishing thus remains prevalent, particularly in places where academic success is measured by the quantity rather than quality of publication output, which feeds the journal’s business model that thrives upon significant market demand. However, such an undesirable enterprise has the potential to flood the scientific literature with unsound research that could be misleadingly perceived as authoritative. This may result in or add to the confusion of policy makers and the layperson, consequentially bringing disrepute to science and all parties involved. Here, we argue that wilfully submitting one’s manuscript to a predatory journal may constitute an active act of avoidance of rigorous peer review of one’s work. If such is the intention, it would be a questionable research practice and could be considered an, albeit covert, form of scientific misconduct. If labelled as such, and with institutional and funding rules erected to discourage the practice, predatory publishing could be effectively put out of business through diminishing the consumer demand.

## Predatory journals and predatory publishing

The advent of open access academic publishing and the shift of the financial burden from reader to author has bred an undesirable side product - predatory publishing (*1*-*3*). Predatory journals and publishers are profit-seeking entities, which pay little attention to, if at all, the quality of academic scholarship. These are most prevailingly found in the form of periodicals and journals, but conferences are also known to be predatory in nature (*4*).

Defining predatory publishing is not straightforward. An authoritative definition recently made by a panel of scholars and publishers in a meeting in Ottawa is: “*Predatory journals and publishers are entities that prioritize self-interest at the expense of scholarship and are characterized by false or misleading information, deviation from best editorial and publication practices, a lack of transparency, and/or the use of aggressive and indiscriminate solicitation practices*” (*5*). Perhaps the most famous attempt in identifying and listing predatory journals and publishers is that by Jeffrey Beall (*6*). Although the original Beall’s list has since been retired, its contents are still being mirrored on websites such as *https://predatoryjournals.com/journals/,* with many others having also weighed in on the defining criteria (*5*, *7*-*10*).

Arguably, the most important criteria that distinguishes predatory journals from authentic scholarly journals is the stringency of peer review (*9*, *11*). A distinguishing feature of authentic journals is therefore a fully functional editorial office and an academic editorial board of a certain quality and commitment. The latter is a requisite in ensuring stringency of peer review, since predatory journals could have fake or non-functional editorial board members (*12*). Without (stringent) peer review, there is essentially no quality control over the content disseminated and this has indeed been proven when publications in a peer reviewed journal were found to have, in general, a higher quality of reporting compared to articles in preprint journals (*13*). Results published in predatory journals thus have no academic stamps of quality and reliability. Many predatory journals may still claim to have a working peer review process, however shoddy.

With the above criteria of “proper peer review” in mind, we might be able to operationally recognize predatory journals based on some prominent superficial features. These would include a combination of active solicitation manuscript from potential authors, a promise of a quick turnaround time from submission to publication, a lack of an established peer review process/system for manuscript review and excessively high article processing charges. Predatory journals tend to canvas for manuscripts by writing to individual authors under the guises of solicitation based on the latter’s reputation often containing glaring errors, such as a failure to properly match the invitee’s expertise to the journal’s scope, or other more trivial discrepancies such as typographical errors. Many, if not most, predatory journals promise an impossibly short turnaround time that are often impractical if the typical process of editorial pre-assessment, reviewer engagement, reviewing timeline and editorial decision making is followed in its entirety. Some predatory journals have no established web-based manuscript submission and processing platform, and authors are instructed to submit manuscripts via email. Of course, the most important point is whether a submitted manuscript has indeed adequate proper peer review by an expert in the field, which has been communicated to the author with the intention of improving the quality of the manuscript. This can be ascertained by readers if the journal publishes reviewer comments with the accepted manuscript, although this is still not widely practiced. However, whether a journal returns quality reviews can be often known by authors in a field or community, even the less experienced junior researchers, based on collegial communications and advisories from mentors. Finally, some predatory journals may have excessively high article publication charges that would allow the publishers to increase their profit margin per publication.

## Prevalence and degradative consequences of predatory publishing

Predatory publishers are increasing in numbers, as are researchers engaging in publishing in predatory journals and conferences. An analysis of 46,000 researchers seeking promotion in Italian academia revealed that about 5% of them have published in journals included in Beall’s list (*14*). For academics in developing countries, the percentages are likely to be even higher for various reasons such as social identity threat, a perception that their research would not qualify for more prestigious, genuine journals and most prominently, the pressure to either ‘publish or perish’ (*15*-*19*). An attractive feature of some predatory journals is the fact that they can be indexed in reputable databases and even acquire an impact factor (*20*-*22*). Although recent analyses suggest that articles in predatory journals are in general poorly cited, ranging from 2.25 to 10 citations per article and therefore have little impact in a field, these articles could nonetheless influence those considered as non-experts (*23*-*26*). This concept of citation contamination, as explored by Anderson, not only propagates potentially flawed and un-vetted science, but also dilutes the impact and importance of genuine, legitimate publications (*20*). The action of citing these articles could be interpreted as being supportive of the predatory journal. The onus is therefore on the authors to ensure that sufficiently in-depth literature analysis is performed to attest to the legitimacy of an article, as well as the journal, prior to citation and indirectly endorsing its contents and the publisher.

While predatory publishing comes in multiple guises, its overall disservice to science and research can be readily predicted (*27*-*29*). For the medical and health sciences in particular, patients could be put at risk by misinformation and errors propagated via predatory journals (*30*). When scientific results that are unreliable become openly accessible to anyone with an internet connection, the public or layperson could be misled into accepting them as valid findings with academic authority. Believing in and acting upon unreliable scientific information, particularly by groups with specific agendas (such as anti-vaccine groups, anti-animal experimentation activists or climate change deniers), could lead to individuals being harmed and societal issues being exacerbated. Even if any such damage is optimistically deemed to be minimal, the waste in financial and manpower resources in producing substandard and useless results would be substantial (*31*). These are amongst some of the ethical issues associated with publishing in a predatory journal (*32*).

## Submission and publishing in predatory journals - reasons and motives

Despite the prevalence and potentially detrimental consequences of predatory publishing, its practice as a business venture with a market demand is difficult to outlaw or extinguish since they operate on a sound business model. There have been a handful of predatory publishers which have been severely sanctioned by the American Federal Trade Commission. Notably OMICS Group Inc, a company engaging in predatory practices was ordered to pay a fine of more than $50.1 million for deceptive practices (*33*). Attempts to increase researchers’ awareness on the pitfalls of predatory publishing may be insufficient, as the market demand continues to exist, and predatory journals become more proficient at masking their true intentions.

Why do researchers submit their work to predatory journals? Some researchers may have submitted and published in predatory journals by mistake or out of ignorance of the latter’s true identity. In a study analysing 300 papers from journals deemed to be predatory in nature, 70% of the corresponding authors were unaware of the true nature of the journals (*18*). These unsuspecting corresponding authors are duped into thinking that predatory journals, who present themselves with a borderline authentic appearance, with sufficient editorial board memberships displayed on their websites, and an apparently well-maintained manuscript submission and review platform are genuine journals. The truly distinguishing feature of these, namely a lack of adequate or stringent peer review, is often unseen unless the process is tested with submissions. The other distinctive feature of predatory journals is the active and personal solicitations for manuscripts. Although these emails are frequently fraught with unmistakable errors indicative of their predatory nature, these invitations can be enticing or coercive, particularly to junior researchers who are appreciative of any attention their work may attract.

In contrast, a good number of researchers, for a variety of reasons, could have actively chosen to submit to illegitimate journals, despite having full knowledge of their predatory nature. These reasons could involve difficulties in getting one’s work accepted in authentic journals, their area of research falling in a niche area where few true experts exists, their research work deemed to be lacking novelty or to not have undergone insufficient depth of analysis due to limited resources or funding.

A very common and prevailing underlying reason for authors submitting to predatory journals is the pressure of career advancement deadlines requiring the establishment of a significant publication track record over a short time (*18*). Indeed, this pressure to publish is prevalent in many countries and fields of research, where the success and productivity of a researcher is often superficially measured by the number of publications, regardless of the calibre of the journal. A review and analysis of the pressures and incentives shaping the decisions by those partaking in predatory publishing has been recently reported (*34*). In this regard, voluntary submissions to predatory journals by researchers essentially constitute the market demand that is keeping the business of predatory publishing going.

## Wilful submission to and publishing in predatory journals – a covert form of research misconduct?

Given that predatory publishing has notable negative impacts on science and research, it follows that the practice of author submission should be actively deterred. This would work only if such submissions and publications are considered undesirable or brought to weigh against the authors’ track record. We ask here if an act of knowingly and wilfully submitting a manuscript for consideration to a predatory journal with the intention of getting a hassle-free, quick addition to one’s publication list constitutes a potential ethical transgression of academic and research norms. Ferris and Winker have reasoned that publishing in predatory journals raise concerns, including *“… issues include misrepresentation; lack of editorial and publishing standards and practices; academic deception; research and funding wasted; lack of archived content; and undermining confidence in research literature”* (*32*). Stein has further invoked Resnik’s four conditions of defining a research misconduct as a measure of whether intentional publishing in a predatory journal is considered a form of scientific misconduct (*35*). Stein surmised that due to the absence of a universally unambiguous definition of predatory publishers, as well as the inability to clearly demarcate the intentionality of publishing in a predatory journal, such an act apparently falls short of being considered a form of scientific misconduct (*36*). However, it would come comfortably under the umbrella of Questionable Research Practices (QRPs), having satisfied the US Institute of Medicine’s two criteria of violating traditional research values, as well as being detrimental to the research process (*37*).

We sought to further explore the possibility of voluntary or wilful submission to a predatory journal (with an established identity as such), being a covert form of scientific misconduct. The classic definition of scientific misconduct categorize acts deviating from proper conduct in research into falsification, fabrication and plagiarism (FFP). According to the US Office of Research Integrity (ORI), research misconduct refers to fabrication, falsification, or plagiarism in proposing, performing, or reviewing research, or in reporting research results. A definition of “falsification” by the U.S. Department of Health and Human Services’ Office of Research Integrity (https://ori.hhs.gov/definition-misconduct) is as follows: “*Falsification is manipulating research materials, equipment, or processes, or changing or omitting data or results such that the research is not accurately represented in the research record”*. The usual interpretation for the term “research record” for researchers would be primary research data recorded in a laboratory notebook or a digital equivalent, which could also be extended to other materials accessible by experts such as description of reagents, equipment and data. However, for the community at large, including policy makers and funders, the primary research data and other forms of materials requiring specific expertise to interpret and appreciate is rather inaccessible and beyond ready comprehension by non-domain experts. To the layperson, “research record” would thus usually mean journal or conference papers whose contents are publicly accessible, with the gist of their contents often further distilled in the form of executive summaries or digests written by scientific journalists.

The key quality assurance step of how research results are eventually disseminated to the public lies primarily in the process of peer review. With adequate peer review, manuscripts with obvious flaws in data acquisition or scientific interpretation are rejected while those with addressable issues are corrected and revised. By wilfully evading proper peer review, yet claiming to have done so, the research record and the steps taken to arrive at its final presentation could hardly be qualified as having been “accurately represented”. It follows that an attempt to deliberately bypass proper peer review would be a manipulation that could result in the possibility of research not being accurately represented. Note that this does not mean that the primary research data would therefore be fraudulent; but rather, the processes undertaken to result in the public presentation of these results, having evaded the quality control step, is deceitful. We thus arrive at the notion that wilfully and knowingly submitting one’s manuscript to a predatory journal could be viewed as an (albeit disguised or covert) attempt at misrepresentation of research. This is a facet of falsification and would thus be a form of scientific misconduct. Notably, in this argument, the motive or intent of the submitting author(s) is of prime importance. Even if there was no initial attempt to falsify or fabricate research data, the intention to get one’s work published through the wilful evasion of stringent peer review, could be construed as research misconduct.

The question then arises as to what constitutes “wilful submission” or “deliberate bypassing of proper peer review”. Operationally, the former would mean that the “predatory” nature of a journal is apparent and known to all authors (since the approval of all authors are required for a submission according to International Committee of Medical Journal Editors criteria), and that submission is voluntary. The latter may include a researcher’s submission to predatory journals as a first choice (as opposed to a compromise made after several rejections by legitimate journals), or be reflected by a high frequency of a researcher’s publication in predatory journals. The former qualifying statement of the predatory identity of a journal being clear is not an attempt to simply remove the variable from the equation. While there are difficulties in ascertaining whether some journals are predatory, there are ample references that would allow any given journal to be adequately assessed and provisionally identified as such. Consultation of senior members of their department, mentors, or institutional officers would also be able to shed light on such a conundrum. Whether there is deliberate bypassing of proper peer review would require some investigation, with evidence produced by the researcher of previous attempts at submission and peer review rejections, or a lack of publication record with predatory journals, could all be mitigating considerations.

We should further qualify that submissions to and publication of manuscripts at a preprint server, where articles are curated but do not undergo peer review, do not constitute a deliberate act of bypassing peer review. It is true that preprints are citable, but most preprints are not subject to peer review prior to online publication and this is made clear to the reader. Furthermore, the preprints may be superseded by the eventual peer reviewed paper. Quite the opposite to being a covert attempt in getting one’s publication record extended without stringent scrutiny, the preprint is an important component of pre-publication peer assessment and transparency in scientific reporting.

## Caveats and rejoinders

The notion above of considering publishing in a predatory journal as a covert form of research misconduct may seem rather radical and unnecessarily harsh. Several objections could immediately be raised. First of all, would this be overly draconic, and would it not be a violation of autonomy, as a researcher should arguably be allowed to freely choose what research topics to work on and where to publish? While this would be idealistically true, undertaking research in the modern setting comes with a responsibility to the public, from which research funding ultimately comes. Presenting inadequately peer reviewed and substandard research would be a waste of tax-payers’ money, spent not just on the research work *per se*, but also in supporting the livelihood of the researcher, let alone if the results are of no use or actually bring harm to the public.

Secondly, one could take issue with the extended interpretation of the “research record” and insist that it should refer to nothing more than the primary research data. In this regard, one’s primary research data could be as accurate as they are obtained, interpreted to the best of the researcher’s ability, and written up for publication; that the manuscript has not been subjected to stringent peer review is not the fault of the researcher and the onus of getting adequate peer review should not be placed upon the author. We would argue that quite the opposite is true. The responsibility of subjecting one’s manuscript to adequate peer review should indeed be placed on the researcher/author, much in the way a researcher should seek ways in ensuring their results are reproducible. In this regard, actively obtaining adequate peer review is no different to one ensuring reproducibility of their data by having analyses repeated by colleagues/co-authors, or key experiments performed in the latter’s presence or with their participation. One could of course further argue that peer review is not a fool-proof manner in identifying instances of falsification or fabrication. As such, peer review would not, on its own, attest to the authenticity and truthfulness of the primary data or the content of a manuscript based on the primary data. In this regard, we agree with Wingfield’s take that “*while the peer review system has flaws, it is still a barrier to bad science”* (*38*). Soliciting proper peer review should therefore be the least one should do before publishing one’s research work. Lapses in performing such due diligence, or worse, deliberately avoiding it, would constitute misrepresentation of research.

Thirdly, one could argue that despite the existence of definitions of predatory publishing and various lists that identify predatory publishers and journals, these definitions could be subjective and controversial. After all, it is said that all publishers are predatory in nature - it is just the extent to which it is predatory that is the question (*39*). Furthermore, as most journal review processes are confidential, how could it be known whether adequate peer review was in fact performed? This is indeed a point that could be difficult to adjudicate. However, with the increasing practice of open peer review, in which reviewers’ names or even the review contents are published together with a paper, the peer review process shall and should become more transparent. The onus should be on publishers and journals to show the research community that they have a stringent peer review process in place.

Finally, should ignorant researchers who publish in a predatory journal “by mistake”, or even those who cite articles published in predatory journals be faulted? Are they not also victims of the “predators” and be considered to have committed an “honest error”, thus exonerating them from any charges of research misconduct, particularly when the career of a junior researcher could be negatively impacted by an innocent mistake like such? We would think that in this case, a clear lack of any “wilfulness” in submission and an intent to deliberately bypass proper peer review would exonerate such authors. However, not knowing enough about the authentic journals in one’s field of work, not having developed an awareness on the issue of predatory publishing and not having done due diligence in assessing a journal prior to submission would mean room for improvement in one’s research acumen.

## Establishing rules against partaking in predatory publishing

If we accept that wilful predatory publishing with deliberate bypassing of proper peer review constitutes a covert act of scientific misconduct on the part of the researcher/author, standardized rules and the corresponding sanctions on research misconduct could then be applied to those who have erred in this regard. The European Federation of academics of sciences and humanities (ALLEA, https://allea.org/) European Code of Conduct for Research Integrity has, under its section 3.1 (Research Misconduct and other Unacceptable Practices), a bullet point of “*Establishing or supporting journals that undermine the quality control of research ‘predatory journals’*”. This does not explicitly spell out “publishing” in predatory journals but could be adopted as such by codes of research integrity in other places. Principally, more research domain specific rules would be fittingly erected by both funding agencies and hosting research institutions. With the former, it could be explicitly stated that manuscripts reporting work arising from the use of grant money should not be submitted to predatory journals. If a case of aberrance occurs, the grant recipient could be barred from further reimbursement of an ongoing grant, or from future grant applications. To the funders, this would be a quality control check to ensure that research money that they are tasked to distribute materialises into scientific results that are reliable and useful.

For the hosting institution, rules could likewise be established, and repeated acts of one publishing in predatory journals could be made to effectively count against their performance assessment especially after adequate warnings against initial transgressions have been served. From the perspective of the hosting institution, this would ensure that research output could be better gauged in terms of quality opposed to quantity, and the reputation of the institution as a whole in research performance would not be tainted by instances of substandard publications. This would follow on from the Hong Kong Principles, which was developed during the World Conference on Research Integrity in 2019 (*40*). This document provides guidance on assessing researchers on their achievements while simultaneously promoting research integrity. In particular, the first principle which states: “*Assess researchers on responsible practices from conception to delivery, including the development of the research idea, research design, methodology, execution, and effective dissemination”*. “Effective dissemination” would be assessed by methods independent of a simple journal article tally, which might include the organisation of public lectures/seminars, or social media metrics. Finkel has also suggested the use of the “rule of five” in assessment of researchers’ publication record, which requires the researcher to qualitatively describe their five most significant publications in the past five years, irrespective of the total number of publications or any associated journal metrics (*41*). Moving the focus from the number of publications, to the quality and significance of these publications would ideally alleviate the pressure to publish which could be considered as the primary reason for wilful submission to predatory journals. Hosting institutions should also ensure that researchers are sufficiently trained to recognise identifying features of predatory publishers and conferences, so that being ignorant of predatory journals would no longer be a mitigating factor.

## Conclusion

In the paragraphs above, we entertain the notion that wilfully submitting and publishing in predatory journals with an intent to bypass proper peer review as a covert form of research misconduct. Thus defined, standard rules against research misconduct could then be brought to bear on the practice author-initiated partaking in predatory publishing, effectively quashing the market demand for the latter. This would help to put an end to the trade that are negatively impacting science and research.
